# The Law Relating to Lunatics and Persons of Unsound Mind.—IV

**Published:** 1903-04-18

**Authors:** J. E. R. Stephens

**Affiliations:** Barrister-at-Law, Author of "Digest of Public Health Cases"


### HOSPITAL ADMINISTRATION.

### CONSTRUCTION AND ECONOMICS.

### THE LAW RELATING TO LUNATICS AND PERSONS OF UNSOUND MIND.—IV.

By J. E. R. Stephens, Barrister-at-Law, Author of "Digest of Public Health Cases."

(*Concluded from page* 449.)

Reception Order by Lunacy Commissioners.

Under section 23 of the Lunacy Act, 1890, any two or more commissioners may visit a pauper lunatic or alleged lunatic not in an institution for lunatics, or workhouse, and may, if they think fit, call in a medical practitioner. If the medical practitioner signs a medical certificate with regard to the lunatic, and the commissioners are satisfied that the pauper is a lunatic, and a proper person to be detained, they may by order direct the lunatic to be received in an institution for lunatics, and the relieving officer of the district or any constable who may by them be required so to do shall forthwith convey the lunatic to such institution.

Lunatics in Workhouses.

Except in the cases mentioned in the Lunacy Act, 1890, no person shall be allowed to remain in a workhouse as a lunatic unless the medical officer of the workhouse certifies in writing: (*a*) that such person is a lunatic, with the grounds for the opinion; and (*b*) that he is a proper person to be allowed to remain in a workhouse as a lunatic; and (*c*) that the accommodation in the workhouse is sufficient for his proper care and treatment, separate from the inmates of the workhouse not lunatics, unless the medical officer certifies that the lunatic's condition is such that it is not necessary for the convenience of the lunatic or of the other inmates that he should be kept separate.

The detention of a lunatic without statutory authority may be justified at common law by showing that it was necessary for his safety or that of others. No lunatic shall be detained against his will or allowed to remain in a workhouse for more than fourteen days from the date of a certificate under section 24 without an order under the hand of a justice having jurisdiction in the place where the workhouse is situate. The order may be made upon the application of a relieving officer of the union to which the workhouse belongs supported by a medical certificate under the hand of a medical practitioner, not being an officer of the workhouse, and by the certificate under the hand of the medical officer of the workhouse.

The form of application may be written or oral, so long as it is supported by the requisite medical certificates. The guardians of the union shall pay such reasonable remuneration as they think fit to the medical practitioner who, not being an officer of the workhouse, examines a person for the purpose of a certificate.

When a pauper lunatic is discharged from an institution for lunatics, and the medical officer of the institution is of opinion that the lunatic has not recovered and is a proper person to be kept in a workhouse as a lunatic, the medical officer shall certify such opinion, and the lunatic may therefore be received and detained against his will in a workhouse without further order if the medical officer of the workhouse certifies in writing that the accommodation in the workhouse is sufficient for the lunatic's proper care and treatment, separate from the inmates of the workhouse not lunatics, or that the lunatic's condition is such that it is not necessary for the convenience of the lunatic, or of the other inmates, that he should be kept separate.

Every reception order expires at the end of one year from its date. The manager of any institution for lunatics, and any person having charge of a single patient, who detains a patient after he has knowledge that the order for his reception has expired is guilty of a misdemeanour.

Reception Orders on Petition.

Subject to the exception in the Lunacy Act, 1890, a person not being a pauper or a lunatic so found by inquisition shall not be received and detained as a lunatic in an institution for lunatics, or as a single patient, unless under a reception order made by a judicial authority. The order shall be obtained upon a private application by petitions accompanied by a statement of particulars and by two medical certificates on separate sheets of paper. The petition shall be presented, if possible, by the husband or wife, or by a relative of the alleged lunatic. If not so presented it shall contain a statement of the reasons why the petition is not so presented, and of the connection of the petitioner with the alleged lunatic, and the circumstances under which he presents the petition. The petitioner shall in the petition undertake that he will personally, or by some one specially appointed by him, visit the patient once at least in every six months.

Upon the presentation of the petition the judicial authority shall consider the allegations in the petition and statement of particulars and the evidence of lunacy appearing by the medical certificates, and whether it is necessary for him personally to see and examine the alleged lunatic; and if he is satisfied that an order may properly be made forthwith, he may make the same accordingly; or, if not so satisfied, he shall appoint as early a time as practicable, not being more than seven days after the presentation of the petition, for the consideration thereof; and he may make such further or other inquiries of or concerning the alleged lunatic as he may think fit. The judicial authority, if not satisfied with the evidence of lunacy appearing by the medical certificates, may, if he thinks it necessary so to do, visit the alleged lunatic at the place where he may happen to be. The petition shall be considered in private, and no one except the petitioner, the alleged lunatic (unless the judicial authority shall in his discretion otherwise order), any one person appointed by the alleged lunatic for that purpose, and the persons signing the medical certificates accompanying the petitions shall, without the leave of the judicial authority, be present at the consideration thereof. Every judicial authority and all persons admitted to be present at the consideration of any petition for a reception order, or otherwise having official cognisance of the fact that a petition has been presented, except the alleged lunatic and the person appointed by the alleged lunatic, shall be bound to keep secret all matters and documents which may come to his or their knowledge by reason thereof, except when required to divulge the same by lawful authority. If the petition is dismissed, the judicial authority shall deliver to the petitioner a statement in writing under his hand, of his reasons for dismissing the same, and shall send a copy of such statement to the commissioners, and shall also, where the alleged lunatic is detained under an urgency order send notice by post or otherwise to the person in whose charge the alleged lunatic is that the petition has been dismissed. Any judicial authority making or refusing a reception order shall, if so required by the commissioners, give to them all such information as they may require as to the circumstances under which the order was made or refused. The powers of the "judicial authority" under the Lunacy Act, 1890, shall be exercised by a justice of the peace specially appointed, or a judge of county courts, or magistrate. The justices of every county and quarter sessions shall annually appoint out of their own body as many fit and proper persons as they may deem necessary to exercise the powers conferred by this Act upon the judicial authority.

HOSPITALS FOR IDIOTS.

The Idiots Act, 1886 (49 & 50 Vict., c. 25), a statute whose provisions were originally included in the Lunacy Act, 1885, but were afterwards embodied in a separate measure, provides for the admission into hospitals, institutions, and licensed houses of idiots and imbeciles, and for their care, education, and training therein. For the purposes of the Act "idiots" or "imbeciles" do not include lunatics. The Act does not extend to Scotland or Ireland. An idiot or imbecile from birth, or from any early age, may, if under age, be placed by his parents or guardians, or by any person undertaking and performing towards him the duty of a parent or guardian, and, until of full age, detained in any hospital, institution, or licensed house registered under the Act for the care of idiots or imbeciles upon the certificate of a medical practitioner that the person to whom the certificate relates is capable of receiving benefit from such hospital, etc., accompanied by a statement of particulars, by the guardians, etc.

